# Melanoma Cells Break Down LPA to Establish Local Gradients That Drive Chemotactic Dispersal

**DOI:** 10.1371/journal.pbio.1001966

**Published:** 2014-10-14

**Authors:** Andrew J. Muinonen-Martin, Olivia Susanto, Qifeng Zhang, Elizabeth Smethurst, William J. Faller, Douwe M. Veltman, Gabriela Kalna, Colin Lindsay, Dorothy C. Bennett, Owen J. Sansom, Robert Herd, Robert Jones, Laura M. Machesky, Michael J. O. Wakelam, David A. Knecht, Robert H. Insall

**Affiliations:** 1 CRUK Beatson Institute, Glasgow, United Kingdom; 2 York Teaching Hospital NHS Foundation Trust, York, United Kingdom; 3 The Leeds Teaching Hospitals NHS Trust, Leeds, United Kingdom; 4 The Babraham Institute, Cambridge, United Kingdom; 5 Beatson West of Scotland Cancer Centre, Glasgow, United Kingdom; 6 Molecular Cell Sciences Research Centre, St. George's, University of London, London, United Kingdom; 7 Alan Lyell Centre for Dermatology, Glasgow, United Kingdom; 8 Department of Molecular and Cell Biology, University of Connecticut, Storrs, Connecticut, United States of America; Yale University, United States of America

## Abstract

The high mortality of melanoma is caused by rapid spread of cancer cells, which occurs unusually early in tumour evolution. Unlike most solid tumours, thickness rather than cytological markers or differentiation is the best guide to metastatic potential. Multiple stimuli that drive melanoma cell migration have been described, but it is not clear which are responsible for invasion, nor if chemotactic gradients exist in real tumours. In a chamber-based assay for melanoma dispersal, we find that cells migrate efficiently away from one another, even in initially homogeneous medium. This dispersal is driven by positive chemotaxis rather than chemorepulsion or contact inhibition. The principal chemoattractant, unexpectedly active across all tumour stages, is the lipid agonist lysophosphatidic acid (LPA) acting through the LPA receptor LPAR1. LPA induces chemotaxis of remarkable accuracy, and is both necessary and sufficient for chemotaxis and invasion in 2-D and 3-D assays. Growth factors, often described as tumour attractants, cause negligible chemotaxis themselves, but potentiate chemotaxis to LPA. Cells rapidly break down LPA present at substantial levels in culture medium and normal skin to generate outward-facing gradients. We measure LPA gradients across the margins of melanomas *in vivo*, confirming the physiological importance of our results. We conclude that LPA chemotaxis provides a strong drive for melanoma cells to invade outwards. Cells create their own gradients by acting as a sink, breaking down locally present LPA, and thus forming a gradient that is low in the tumour and high in the surrounding areas. The key step is not acquisition of sensitivity to the chemoattractant, but rather the tumour growing to break down enough LPA to form a gradient. Thus the stimulus that drives cell dispersal is not the presence of LPA itself, but the self-generated, outward-directed gradient.

## Abstract 2

Melanoma cells break down lysophosphatidic acid from the environment, creating a chemotactic gradient that the tumor cells then follow; this provides an explanation for the rapid metastasis of melanoma.

## Author Summary

Melanoma is feared because it spreads very rapidly when tumours are relatively small. It is not known why this metastasis is so efficient and aggressive. In particular, it is not known what drives melanoma cells to start to migrate out from the tumour. Here, we have studied the chemical signals that guide the migration of melanoma cells. We find that a component of serum, lysophosphatidic acid (LPA), functions as a remarkably strong attractant for all of the melanoma cells that we examined. We also observe that melanoma cells rapidly break down LPA. We conclude that melanomas create their own gradients of LPA, with low LPA in the tumour and high LPA outside. Since melanoma cells are attracted by LPA, this LPA gradient around the melanomas serves as a signal that drives the tumour cells out into the surrounding skin and blood vessels. Finally, we show that such gradients exist in a mouse model of melanoma. Self-generated LPA gradients are therefore an intriguing new driver for melanoma dispersal.

## Introduction

### Melanoma Metastasis

Melanoma is an unusually aggressive cancer, which often metastasizes early during tumour development [1]. Tumours that have not clinically metastasized are frequently curable, but patients are far less likely to survive if tumours have metastasized before they are surgically removed, and metastasis is the principal cause of cancer mortality [2]. The most influential prognostic factor in predicting metastasis and survival is the thickness of the tumour (the “Breslow depth”) [3]. There is a dramatic increase in the risk of metastasis with only millimeter increases in Breslow depth [3]. This characteristic is unlike most solid tumours, in which the cytological morphology of the tumour cells and the individual genes mutated in the cancer are more important than size alone. Metastasis is therefore an important, and undermedicated, potential target for cancer therapy [4],[5].

### Melanocyte Migration during Development

One principal reason behind the aggressiveness of melanoma derives from the developmental history of melanocytes, the pigment producing cells in the skin that mutate to form melanomas. During mammalian development melanoblasts, the melanocyte precursors, emerge from a restricted location at the neural crest, and migrate rapidly from there throughout the developing dermis, before maturing into melanocytes on the basement membrane of the epidermis [6]. Thus a substantial level of cell migration is required for even skin pigmentation. Even in adults—for example following treatment for vitiligo—melanocytes can spread significant distances from the hair follicles to repopulate the surrounding skin. The melanocyte lineage is thus inherently migratory.

**Figure 1:**
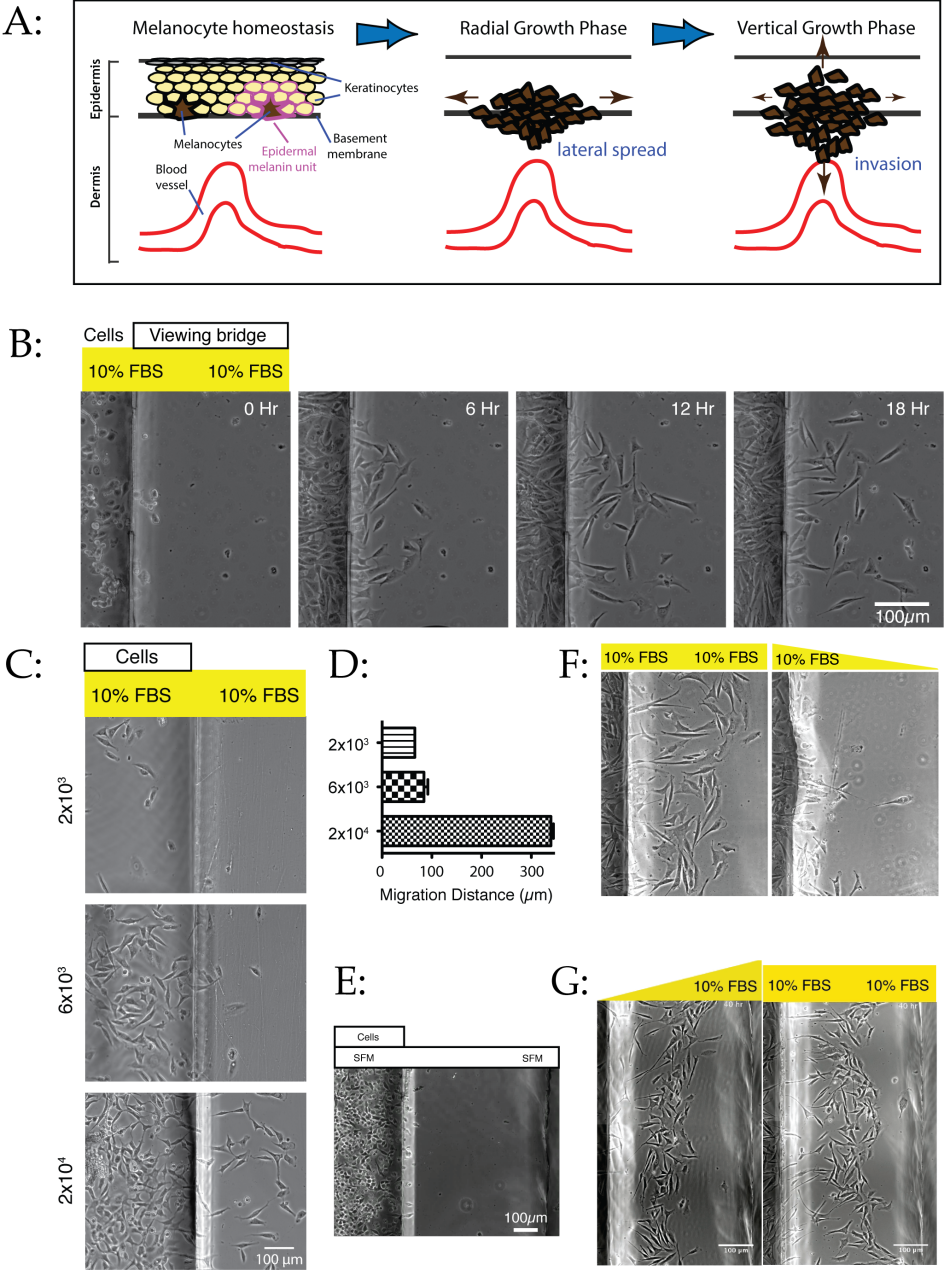
Density-dependent dispersal of melanoma cells. (A) Schematic showing the stages of melanoma spread. (B) WM239A metastatic melanoma cells dispersing in uniform medium. 2×10^4^ cells were introduced into one reservoir of an Insall chamber containing complete medium with 10% FBS throughout, and observed by time-lapse phase contrast microscopy. See Movie S1. The left side of each image shows the reservoir containing cells, while the right side is the viewing bridge of the chamber. (C–D) Migration is density-dependent. WM1158 metastatic melanoma cells were seeded at different densities in full medium with 10% FBS, and observed as before. At 2×10^4^ cells/well and above, peak migration distances increase sharply, as confirmed by the distance at 17 hours (D; graph shows mean ± SEM). (E) Migration is not driven by production of a repellent. 2×10^4^ WM1158 cells were introduced into a chamber in minimal medium without serum and observed at 17 hours as before. Cells survive and adhere, but do not disperse. (F) Migration is not driven by production of a serum-derived repellent. 2×10^4^ WM1158 cells were introduced into a chamber in minimal medium without serum and observed at 17 hours as before. Cells disperse less efficiently in conditioned medium than in fresh medium. (G) Migration mediated by chemotaxis up a serum gradient is similar to density-induced migration. Left panel: 2×10^4^ WM1158 cells were introduced into a chamber in the presence of a gradient from 0% FBS around the cells to 10% in the opposite reservoir [15]. The cells rapidly migrate towards the well containing serum. Right panel: similar assay with 10% serum in both reservoirs. Panels taken from Movies S3 and S1, respectively.

However, several questions about melanoma progression remain unanswered. The first is what drives melanomas to change from the relatively benign radial growth phase (RGP) to the far more invasive vertical growth phase (VGP) (see schematic diagram in Figure 1A). In RGP melanomas, cells only spread horizontally along the basement membrane, compared to VGP melanoma cells, which are also capable of spreading both upwards into the epidermis (Pagetoid spread) and downwards, into and through the dermis (invasion). This spread raises the related question, of what drives cells to migrate away from the primary tumour. Simple, random migration is an extremely inefficient way of dispersing cells and also unlikely to drive cells to invade through matrix and basement membranes. Chemotaxis—cell migration directed by gradients of soluble signalling molecules—is implicated as an important driver of metastasis by a wide range of data [7],[8], and is considered necessary to drive efficient invasion. In breast cancer, for example, some tumour cells migrate towards epidermal growth factor (EGF) [9]. However, EGF gradients have only been inferred *in vivo*, never measured, and their sources are usually unclear. In the case of breast cancer, the EGF is thought to be secreted by macrophages recruited in a paracrine loop by the tumour [10], but for other attractants and cell types the sources of chemotactic signals are not known.

In the melanoma literature, most chemotaxis is attributed to growth factors such as platelet-derived growth factor (PDGF) and EGF [11] and the CXCR4 ligand SDF-1 [12], though a wide variety of potential attractants have been discussed [13]. Gradients of growth factor or SDF-1 have not been identified *in vivo*, they can only be inferred from the cells' behaviour or pattern of responses *in vitro*.

### Chemotaxis and Invasion Assays

Chemotaxis assays are typically performed in transwell chambers, in which cells are grown on one side of a membrane filter and potential attractants are added to the other side. Chemotaxis is assayed by the number of cells observed on the far side of the filter after a fixed interval. These assays are subject to a wide range of artifacts. Cells' behaviour during chemotaxis cannot be studied, which makes it extremely difficult to distinguish chemotaxis from directionless changes in migratory behaviour (i.e., chemokinesis [14]). Potential attractants form extremely steep and rather short-lived concentration gradients, unlike the physiological conditions the assay aims to reproduce. More seriously still, conditions either side of the filter may be discretely different; cells may grow, survive, or adhere better on one side of the filter than the other, giving changes in the numbers of cells that can be artifactually interpreted as chemotaxis. Direct viewing chambers, such as Dunn, Zigmond, or Insall chambers, are more laborious to use but yield a far higher quality of data, with fewer artifacts [15]–[17]. In work described here, we use direct-viewing chambers to identify lysophosphatidic acid (LPA) as a far more potent chemoattractant for melanoma cells than other previously described attractants. We have developed and refined two direct-viewing assays to assess mechanisms of cell dispersal and chemotaxis, allowing us to distinguish chemotactic from chemokinetic and contact-driven responses under defined conditions that minimize artifacts. Furthermore, the use of direct-viewing chambers makes comparison of attractants' relative efficiencies practical.

### The Source of Attractant Gradients *In Vivo*

The suggested role of chemoattractants in cancer dispersal—whether growth factors, chemokines, or LPA—raises the crucial question of how gradients are generated. Chemotaxis will only work with signals that are presented as gradients—homogeneous signals contain no directional information—and the steeper the gradient, the more efficient the chemotaxis. Chemical gradients are typically effective over distances of less than a millimetre—limits on the efficiency of diffusion make larger gradients impractical [18]. Thus for a gradient to be formed there must be a gradient source that is close to the tumour.

Alternatively, local gradients may be formed from signals that are widely produced, but are absorbed or broken down locally. This local depletion mechanism is potentially just as effective as local production, but less often invoked. In the cancer literature, only localised sources are typically invoked, for example individual macrophages within the vasculature attracting cancer cells within the tumour [10].

If cells that are responding to a stimulus are also responsible for breaking it down, the result is a self-generated gradient. Under these conditions the gradient is always oriented away from the current location of the cells. One such example has been shown during the development of the zebrafish lateral line primordium [19]–[21], in which a dummy receptor locally absorbs an SDF-1 stimulus to set up a gradient that is detected by a different receptor. In this work we find that melanoma cells self-generate chemotactic gradients from unlocalised, exogenous LPA. These gradients tend to direct cells to disperse outwards from tumours, thus directly promoting metastasis. Furthermore, we measure LPA gradients across real melanomas *in vivo*. Since melanomas of sufficient size both generate their own LPA gradients and respond to them, chemotaxis-steered spread of melanomas is almost inevitable.

## Results

### Density-Dependent Outward Migration of Tumour Cell

To examine the signals that drive the spread of melanoma cells, we set up 2-D assays for tumour cell spread using a direct-viewing chemotaxis chamber that allows detailed analysis of cell migration [15]. The chamber contains two wells, connected by a bridge that allows diffusion of attractants but not flow. Both cells were homogeneously filled with complete medium, but cultured melanoma cells [22] were only seeded in one well, at a range of different densities.

Our initial results were surprising: Cells consistently spread outwards from the well in which they started, even in uniform medium without an externally applied gradient (Figure 1B; Movie S1). This effect was density-dependent; cells plated at 2×10^3^ or 6×10^3^ cells/well barely migrated, while 2×10^4^ cells/well migrated up to 350 µm in 24 hours (Figure 1C and 1D). This behaviour strikingly resembles the behaviour of real melanomas, in which the chance of metastasis is more correlated with tumour thickness than any other parameter [3].

This type of density-dependent spreading requires individual cells (or small clusters of cells) to migrate away from the bulk population. This dispersal occurred in our assays; cells moved directly away from the well they resided in with unprecedented accuracy (Movie S1). This directed, non-random migration can only occur if the moving cells perceive a directional cue from the bulk population of the cells to spread. We therefore analyzed the nature of the signal that was directing cells away from the bulk population. The most probable signalling mechanisms are contact inhibition of migration [23] or chemotaxis. We therefore examined these potential mechanisms in turn.

Contact inhibition (of migration, as opposed to the more frequently described contact inhibition of growth) is an effective mechanism for short-range dispersal in which cell∶cell contact directs cells away from one another. It has been shown in other neural crest-derived cell types [24]. However we found no evidence to suggest it drives cell dispersal in our assays. Movie S2 shows one example in which cells spread both individually and while contacting one another. Some cells steer accurately outwards through multiple cycles of new pseudopods independently of cell∶cell contact. Others continue to migrate outwards when contacting the cell in front, where contact inhibition predicts these cells should reverse into the space behind them. Analysis of the paths of individual cells (Figure S1) shows that cell-cell contact is not steering cells; the paths of cells that are contacting others, have recently contacted others, and are not in contact are strikingly similar. The one apparent example of contact inhibition (Movie S2, cell 2) changed the cell's direction but did not improve its outward accuracy. Thus while these cells may experience contact inhibition, we considered chemotaxis as the most likely mechanism steering them away from the main population.

Cells could generate chemotactic gradients to drive dispersal by either of two mechanisms. They could secrete an autocrine chemorepellent and migrate away from it. We have previously shown this to be a key driver of Entamoeba pathogenesis [25], in which chemotaxis away from ethanol generated by the amoebas themselves causes cells to migrate from the lumen of the gut into the walls of the gut and eventually the liver of the patient. Alternatively, the melanoma cells could locally break down or consume a chemoattractant that is produced externally, but spatially homogeneously [26],[27], as seen in the zebrafish lateral line primordium [19],[21]. In either case, dense populations of cells create a gradient that consistently directs migration away from themselves. We considered that homogeneous attractants would most likely derive from the serum added to full medium. To find if dispersal used a repellent or a consumed attractant, we compared cell dispersal in serum-free and normal medium. Cells in serum-free medium are healthy and motile in control movies, but do not migrate away from one another (Figure 1E), demonstrating that the cells do not secrete chemorepellents. We also compared cells moving out of fresh medium into serum-free and full medium. Cells dispersed far more efficiently into the rich medium (Figure 1F), implying that they are driven by attractants in fresh medium rather than an inhibitor whose production depends on serum.

To test whether consumption of a component of serum produces a positive chemotaxis response, we compared migration in uniform serum to an assay in which cells are exposed to a gradient between serum-free medium and medium supplemented with 10% serum (Movie S3). We found that both assays produced similar directed migratory responses; cells migrated towards the opposite well with or without a preformed serum gradient (Figure 1G). This finding further supports the concept that the outward migration is driven by positive chemotaxis, most likely towards a chemoattractant globally present in the serum but depleted around the cells.

**Figure 2:**
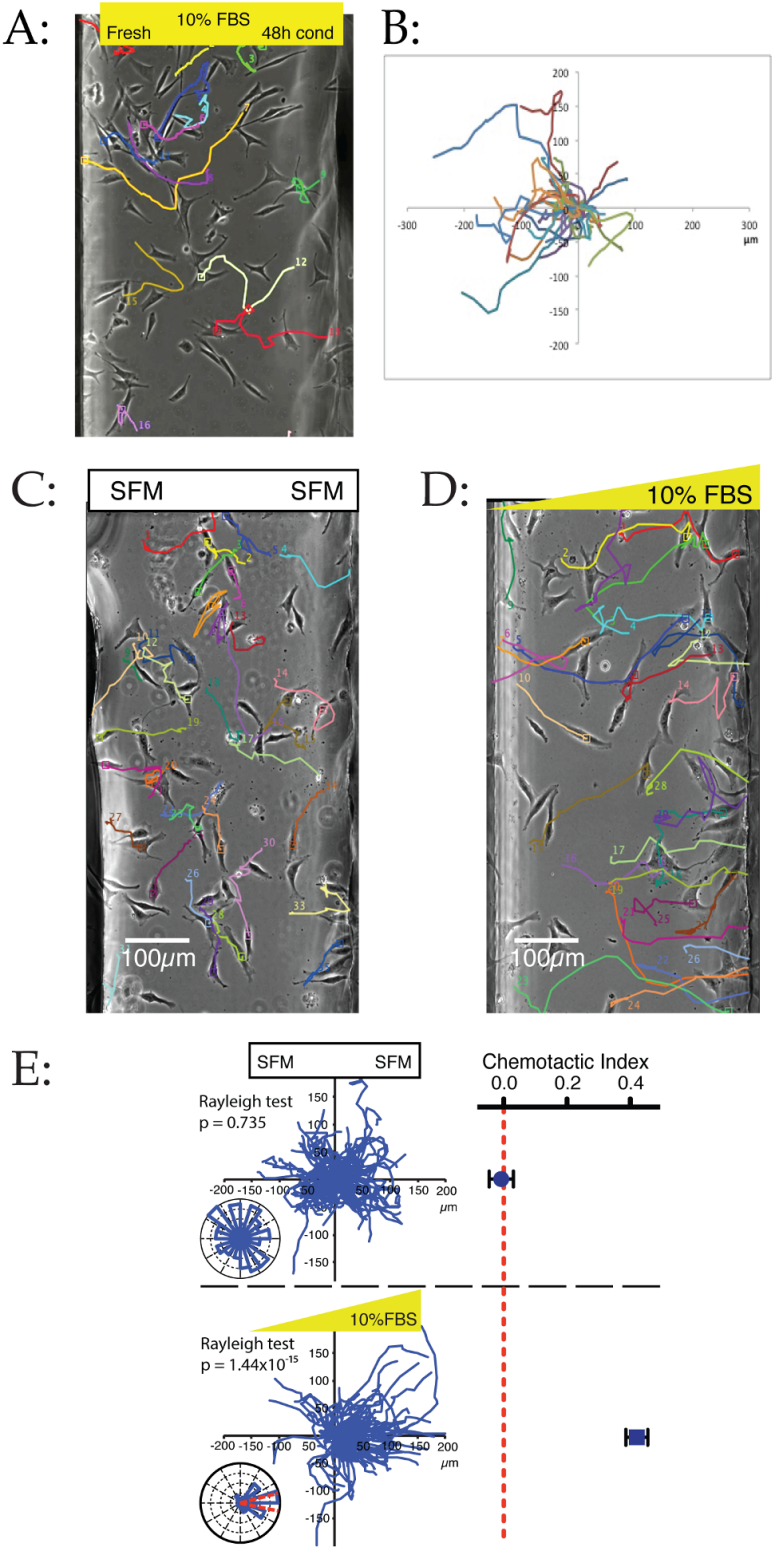
Dispersal is due to a chemoattractant present in serum. All panels show data from melanoma cells migrating in chemotaxis chambers as described [15]. (**A–B**) Cells migrate from conditioned medium towards fresh medium. WM1158 cells were randomly attached to a coverslip and assembled in a chamber in 48 hour WM1158 cell conditioned medium. The medium in one chamber was replaced with fresh medium, while the other was left alone. Tracks of individual cells are shown as coloured lines (A). Cells move towards the fresh medium, as shown by the spider plot (B) showing all cell tracks. (**C–D**) Example images showing WM239A metastatic melanoma cells after 21 hours in serum-free medium (C) and a 0%–10% FBS gradient (D). Coloured paths show centroid tracks from time 0. (**E**) Quantitative analysis of chemotactic responses. “Spider” plots (large panels), rose plots, mean chemotactic index, and Rayleigh test for directionality are shown for cells in serum-free medium and a 0%–10% FBS gradient (*n*>100 cells in three independent experiments for both conditions). Spider plots show strong chemotaxis in FBS gradients; in serum-free medium only random movement is seen. Rose plots show overall movement from 6–12 hours; the proportion of total cells in each sector is shown on a log scale, with red lines representing the 95% confidence interval. The majority of cells in the FBS gradient move in the direction of the chemoattractant. Rayleigh tests statistically confirmed this highly significant unimodal directionality. Graphs of chemotactic index were generated from the same data.

We tested this hypothesis using a more traditional chemotaxis assay, in which cells are spread homogeneously over the field at the start of the assay, giving the cells the opportunity to move in any direction [14]. We loaded cells into the chamber in complete medium that had been conditioned by melanoma cells for 48 hours, then replaced the medium in one well with fresh medium containing 10% serum. The cells migrated towards the well containing fresh medium very efficiently (Figure 2A and 2B), showing that an attractant in fresh medium is consumed by the melanoma cells.

We confirmed that chemoattractants are present in normal serum by exposing melanoma cells—again homogeneously seeded in the chemotaxis chamber—to exogenous gradients of serum. In homogeneous serum-free medium the cells were healthy, and migrated, but randomly (Figure 2C). When a gradient of serum was applied, the cells migrated towards the higher concentrations with unprecedented precision (Figure 2D); their paths are overwhelmingly oriented up-gradient, in a manner more usually associated with neutrophils and Dictyostelium [28] than cancer cells, which typically chemotax less accurately [29]. The high chemotactic index was maintained throughout a sustained period, with narrow and accurate confidence interval, and strongly significant Rayleigh test [30] for directional migration (Figure 2E). Thus serum contains a remarkably potent chemoattractant for melanoma cells.

We therefore conclude that melanoma dispersal across the chamber is driven by positive chemotaxis towards an attractant that is present in serum. The attractant is broken down by the cells themselves into a gradient that efficiently disperses cells.

### Chemotaxis during Tumour Progression

**Figure 3:**
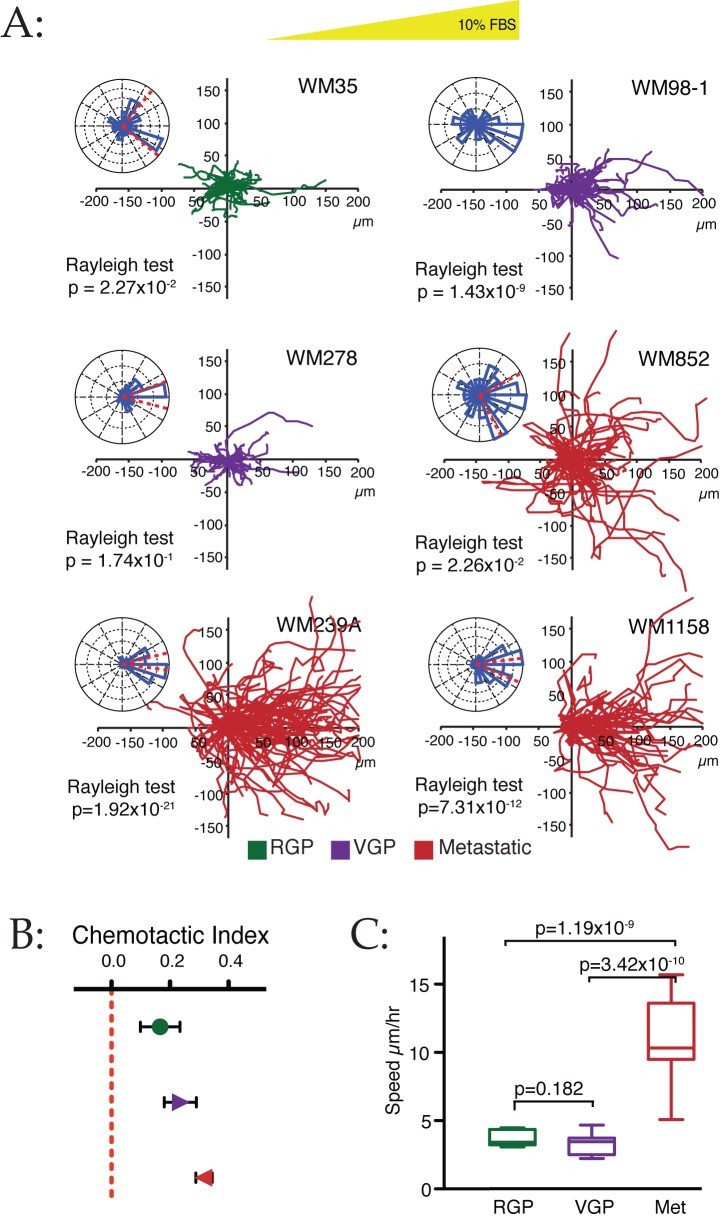
Chemotaxis of cells from different melanoma stages. (A) Chemotaxis of a panel of six cell lines from different melanoma stages (RGP, green; VGP, purple; metastatic, red) up a 0%–10% FBS gradient was measured as above (*n*≥45 cells per cell line). (B) Chemotactic index of cells from different stages. Data from (A) were collated by melanoma stage. Chemotaxis improves as the stage of melanoma progresses, although even the earliest RGP cells show clear chemotaxis. (C) Speeds of cells from different stages. Data from (A) were collated by melanoma stage. Metastatic lines are conspicuously faster (*p*-values from unpaired *t*-tests), although again the speed of RGP and VGP cells is still relatively high for non-haematopoietic cells.

One potential explanation for cancer cells becoming metastatic is that they evolve chemotactic competence as the tumours develop [13],[31],[32], and thus move from unsteered to steered migration. We therefore examined the ability of a panel of cell lines isolated from different tumour stages and selected for physiologically appropriate behaviour (Figure 3A) [22]. Surprisingly, all the lines we examined responded chemotactically to serum gradients (Figure 3B). Cells from metastases were more motile than cells from earlier stages (Figure 3C); highly invasive (VGP) cells were slightly more accurate, but not significantly faster than the biologically earlier, RGP cells. Cells from more advanced tumours responded more robustly, but the progression from nonmetastatic to metastatic was not marked by the cells newly acquiring responsiveness—all lines examined were chemotactic enough to spread away from the tumour efficiently in the presence of an appropriate gradient. Several lines of data suggest that genetic and epigenetic changes during progression from RGP to VGP increase cells' ability to survive [33]; our data imply that it is cell survival, rather than chemotactic sensitivity, that defines the difference. The increase in migratory ability could modulate cells' ability to escape from a primary tumour, but our principal conclusion is that melanoma cells from all stages are chemotactic.

### Identifying the Chemoattractant in Serum

**Figure 4:**
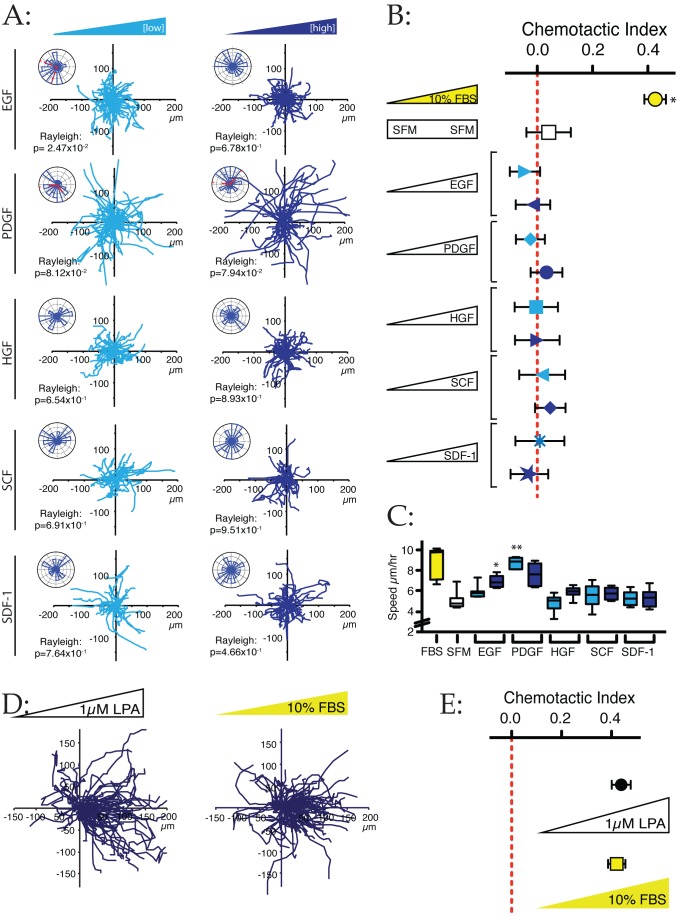
Identification of LPA, rather than growth factors, as the principal attractant in serum. (A) WM239A cells were exposed to gradients of low (light) and high (dark) concentrations of several growth factors and the chemokine SDF-1 in combination with SFM. Spider and Rose plots with Rayleigh tests are shown (*n*>40 cells for each condition). Concentrations tested were EGF (6.25 and 25 ng/ml), PDGF (25 and 100 ng/ml), HGF (10 and 30 ng/ml), SCF (10 and 100 ng/ml), and SDF-1 (100 and 300 ng/ml). None shows obvious chemotaxis. (B) Quantification of data from (A). Serum gradients promote strong chemotaxis (*p*<0.0001, unpaired *t*-test), but gradients of all growth factors tested show no significant chemotactic index (*p*≥0.40). (C) Growth factors enhance cell speed. Data quantitated from the cells in Figure 3A. Directionless cell speed was measured by totalling the distance moved between time points. EGF and PDGF stimulate cells in serum-free minimal medium to speeds comparable with serum gradients. Single asterisk: Different from SFM alone, *p*<0.001, unpaired *t*-test; double asterisk: *p*<0.0001). (D) LPA and serum drive comparably efficient chemotaxis. WM239A cells were examined in a chamber responding to 0%–10% FBS and 0–1 µM LPA. The spider plot shows similar cellular responses to the two gradients. (E) Quantitative analysis of chemotaxis towards LPA and serum. Chemotactic index was calculated from three experiments including that shown in (D). Cells respond comparably to both conditions. Bars show SEM.

There are multiple reports of chemotaxis driving metastasis of melanoma and other tumour cells, in particular breast cancer. Published accounts of chemotactic invasion most often describe growth factors as the attractants—for example EGF for solid tumours [34], and EGF, hepatocyte growth factor (HGF), and stem cell factor (SCF)/KitL for melanoma [13]. However these attractants were often identified in transwell chambers, which as earlier discussed are subject to a range of artifacts, in particular false positive. For example, the positive well might promote survival, growth, or adhesion of cells that move randomly across the membrane. Our direct-viewing chambers provide a far more rigorous analysis. We therefore tested a broad range of attractants in our assays. To our surprise, no growth factor acted as an attractant to any measurable degree (Figure 4A); steep or shallow gradients gave no obvious movement upgradient, and no significant chemotactic index towards any growth factor tested (Figure 4B). We therefore conclude that the chemotaxis towards serum we observed was unlikely to be towards growth factors. This does not, of course, demonstrate that melanoma cells are never chemotactic towards growth factors; but it clearly shows the surprising and efficient chemotaxis towards serum observed earlier is mediated by another molecule.

EGF and PDGF did increase cells' speed (Figure 4C), but they did not provide directional specificity. They therefore acted as chemokines, regulating overall cell behaviour, rather than as chemoattractants that could steer the cells.

The striking accuracy of chemotaxis demonstrated by melanoma cells towards serum was more reminiscent of neutrophil chemotaxis towards formyl peptides, or *Dictyostelium* towards cAMP, which signal through G-protein coupled receptors (GPCRs) rather than growth factor receptors like EGFR and PDGFR. We therefore investigated SDF-1, the ligand for the GPCR CXCR4, which has been associated with poor prognosis and malignancy of melanoma [35]; but again, it was not measurably attractive to cells in our assays (Figure 4B, compare with strong response to serum).

However, LPA, another well-known component of serum that signals through GPCRs, was strikingly attractive to melanoma cells. A gradient from 0 to 1 µM LPA across the chamber (consistent with the approximate concentration of LPA in serum; see below) induced chemotaxis almost as effectively as 0%–10% serum (Figure 4D), yielding a comparable chemotactic index (Figure 4E). This was a surprise: LPA is more typically described as an inflammatory mitogen, acting on haematopoietic cells such as macrophages. It appears frequently in the cancer literature, but more often as a mitogen and chemokine for cancer cells, acting via autotaxin, which catalyzes the production of LPA from lysophosphatidylcholine [36]. However in our assays the chemotaxis of melanoma to LPA was again remarkably accurate compared with the weaker chemotaxis typically seen in cancer cells [37].

### LPA Is the Dominant Attractant in Serum in 2-D and 3-D Assays

**Figure 5:**
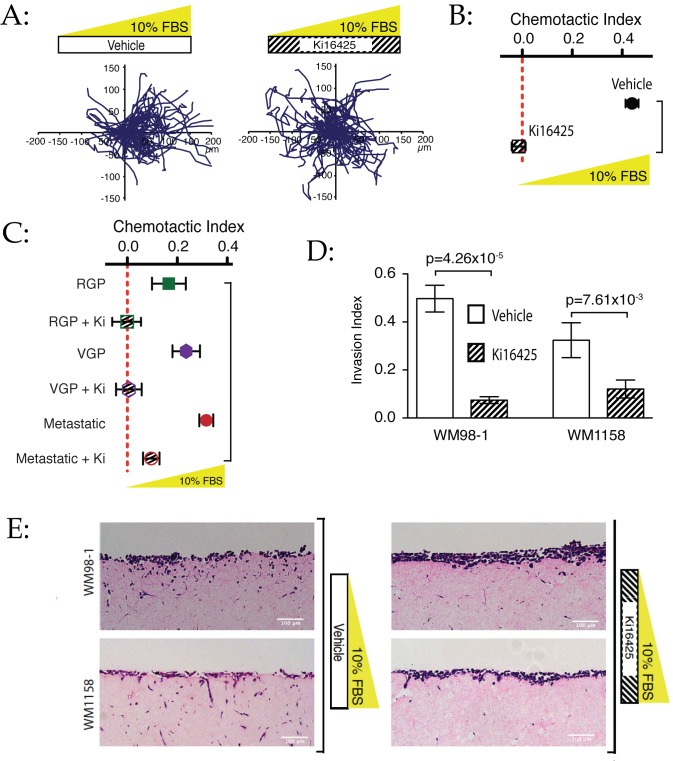
LPA responses are essential for serum chemotaxis in 2-D and 3-D assays. (A) LPA receptor antagonist Ki16425 blocks chemotaxis to serum. Chemotaxis of WM239A cells was compared with and without 10 µM Ki16425. Inhibitor-treated cells showed no chemotaxis despite essentially normal random migration. (B) Quantitative analysis of Ki16425 activity. Data from three experiments, including the one in (A). The chemotactic index of inhibitor-treated cells is essentially zero. (C) Melanoma cell lines from all stages chemotaxing up a 10% serum gradient with and without Ki16425. Colours represent melanoma stage. In RGP and VGP cells, chemotaxis is totally blocked, while in metastatic lines it is substantially inhibited. Bars show SEM. (D–E) 3-D organotypic assays. The cell lines WM98-1 and WM1158 are shown ±Ki16425. LPA receptor antagonist greatly inhibits invasion. In (D), invasion index is calculated as the percentage of total cells on the organotypic matrix that invaded beyond ∼30 µm as a ratio of cells on top of the matrix (*n*>1,000 cells per condition). (E) shows haematoxylin and eosin-stained vertical sections through gels, showing downward invasion of melanoma cells.

To examine whether LPA was the principal attractive component of serum, we assayed chemotaxis in the presence of the antagonist Ki16425, which specifically inhibits binding to LPA receptors 1 and 3 [38]. The effects were again remarkably clear. 10 µM Ki16425 blocked cell spread in our original, density-dependent assay (Movie S4) and chemotaxis towards 10% serum (Figure 5A; Movie S5), reducing the chemotactic index from more than +0.4 to zero (Figure 5B). Ki16425-treated cells were obviously healthy, and moved similarly to untreated cells, with similar track lengths, showing that the treatment was not making the cells nonspecifically sick or non-motile. Knockdown of LPAR1 by siRNA had a similar effect (Figure S2A), showing that LPAR1 is the key receptor for this process, and 10 µM Ki16425 also blocked chemotaxis towards pure LPA (Figure S2B). Again, LPA chemotaxis is not tumour stage-specific; Ki16425 blocked chemotaxis in all cell lines from all stages of cancer progression (Figure 5C). RGP and VGP cell lines were completely inhibited, and the highly motile metastatic lines were substantially inhibited. The residual chemotaxis in the presence of inhibitor could represent either incomplete inhibition by the antagonist, or a small amount of chemotaxis to another agent. From these data, we conclude that LPA is overwhelmingly the dominant chemoattractant in serum for all lines examined.

While chamber-based assays are optimized to allow accurate and detailed recording, they provide a 2-D view of a process that more often happens in 3-D tissues *in vivo* [39]. We therefore examined the role of LPA in a widely used organotypic tumour cell invasion model [40]. In this system melanoma cells are added to the top of a plug of collagen in which fibroblasts are growing, and over time they migrate vertically downwards into the 3-D matrix. During the course of the assay, the collagen plug is set so only its bottom face contacts the medium, at which point malignant melanoma cells invade downwards [41]. We hypothesized that the melanoma cells were driven by a self-generated LPA gradient as in Figure 1B, once fresh LPA could only be supplied from the bottom. This hypothesis is supported by assays in which the collagen plugs remain submerged, and no invasion is seen (Figure S3), further rejecting contact inhibition of migration as a mechanism of invasion. When the gels were treated with Ki16425, the melanoma cells did not invade downwards into the gel (despite comparable numbers of cells at the end, showing no change in growth or survival). Quantitative analysis confirms that Ki16425 strongly inhibited invasion in both cell lines that were invasive in this assay (Figure 5D and 5E). Thus LPA is a dominant steering system for 3-D organotypic assays, as well as for 2-D chamber assays.

### Melanoma Cells Break down LPA to Form Outward-Facing Gradients

**Figure 6:**
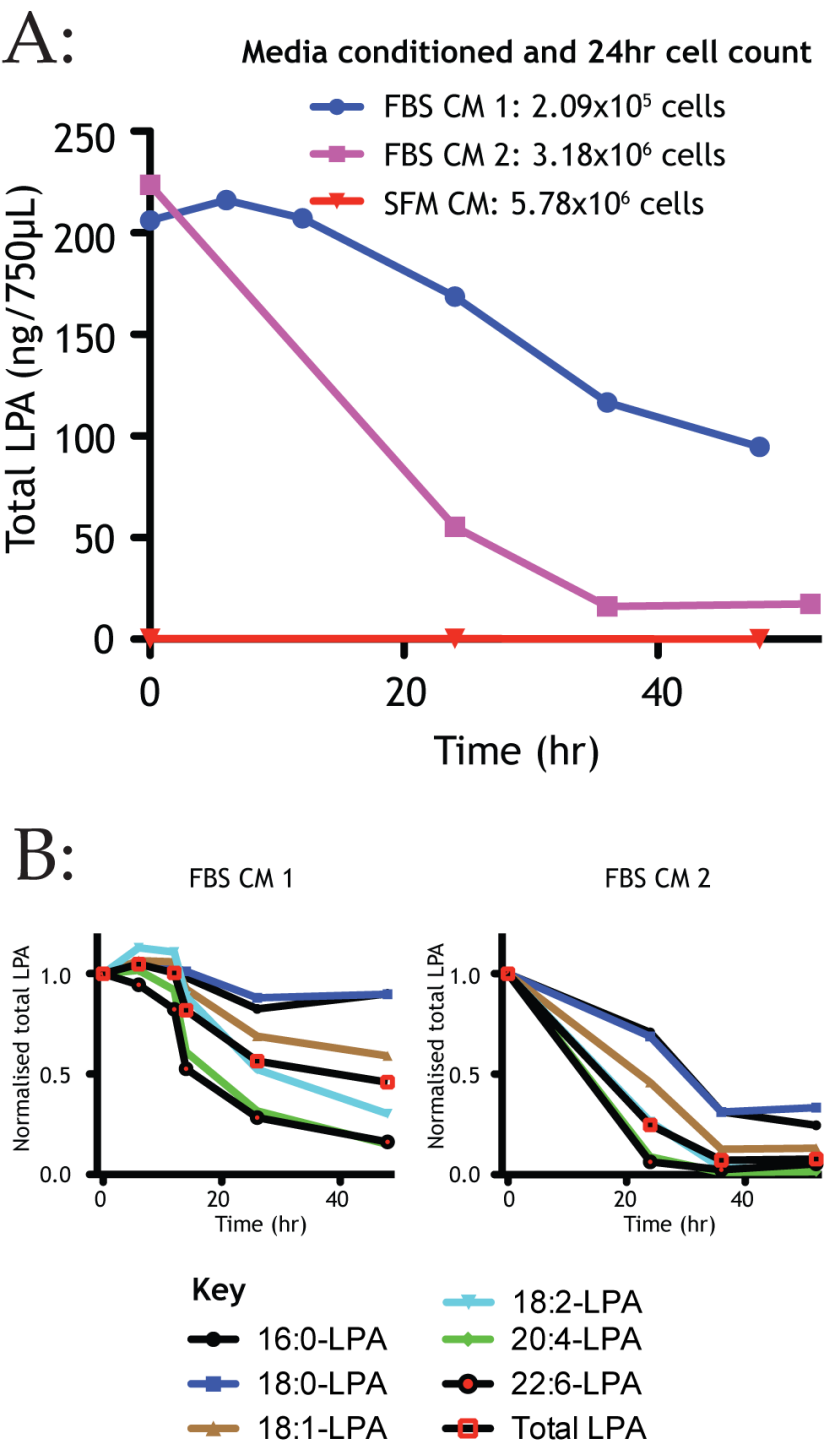
Melanoma cells preferentially break down signalling forms of LPA. (A) LPA concentration over 48 hours during conditioning of media, both with and without 10% FBS by melanoma cells (WM239A). FBS conditioned media demonstrates density-dependent depletion of LPA as measured by mass spectrometry. LPA remained negligible throughout 48 hours of serum-free conditioning by the same cells. Representative graph. (B) Analysis of LPA subspecies during melanoma cell conditioning demonstrates bioactive isoforms were depleted more rapidly by melanoma cells in both samples. Two representative graphs are shown to illustrate quantitative variability but qualitative consistency.

Our earlier data (Figures 1B and 2A, in particular) showed that melanoma cells disperse by depleting a chemoattractant from serum. We therefore tested whether melanoma cells are able to deplete LPA from their surroundings. Full medium with and without serum was incubated with different densities of melanoma cells for different times, then LPA was extracted from the conditioned medium and analyzed by mass spectrometry [42]. This confirms that the melanoma cells effectively break down LPA; the conditioned medium was depleted in a density-dependent manner (Figure 6A) and in a timescale that correlates with the medium conditioning experiments in Figure 2A and 2B. One advantage of using mass spectrometry is the identification of molecular subspecies. The biological activity of LPA is known to vary with its structure [43],[44]. In particular, there is a strong correlation between biological activity and the degree of polyunsaturation, and also acyl chain length [45]. Melanoma cells broke down the biologically active species more rapidly than the others (Figure 6B), ensuring that the most active species also formed the steepest gradients.

### The Role of Growth Factors

**Figure 7:**
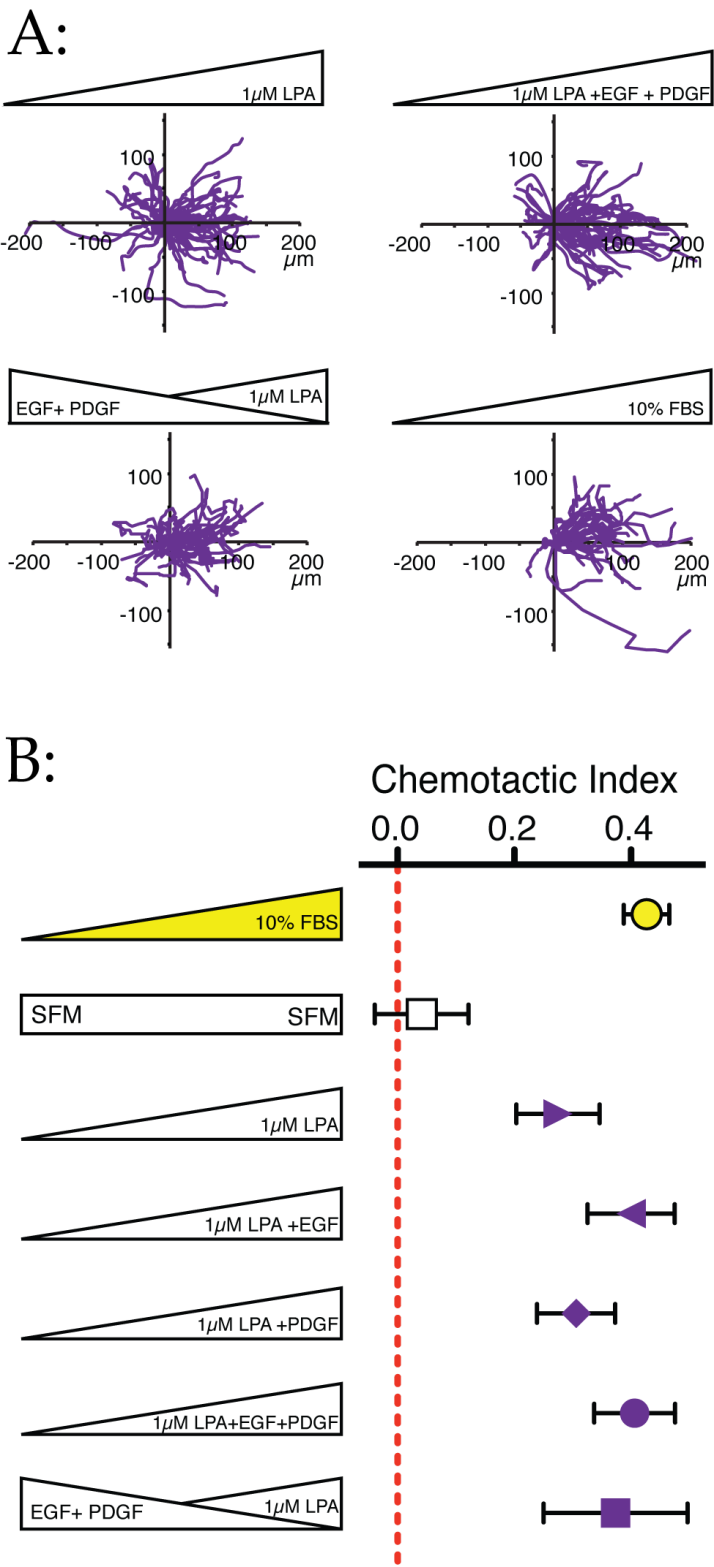
Growth factors potentiate LPA chemotaxis. (A) Growth factors enhance cells' response to LPA gradients. Figure shows plots of the WM239A paths chemotaxing in gradients of LPA, LPA+EGF+PDGF, and conflicting gradients of LPA versus EGF+PDGF. (B) Chemotactic indices of cells in (A) and other conditions. Growth factor gradients if anything increase the efficiency of LPA chemotaxis, even when applied in a gradient in the opposite direction. Bars show SEM.

The results we have obtained conflict with the established dogma that growth factors are primary melanoma chemoattractants [13]. To reconcile these accounts with our data, we examined the role of growth factors during chemotaxis towards LPA. As shown previously (Figure 4C), EGF and (particularly) PDGF increased the basal speed of cells. Gradients of EGF and PDGF, and mixtures of both, enhanced the accuracy of chemotaxis to LPA (Figure 7); LPA, EGF, and PDGF together in serum-free minimal medium were as effective as 10% serum. Most tellingly, however, when cells were presented with LPA and growth factor gradients oriented in opposite directions, they chemotaxed towards the LPA not the growth factors; if anything they migrated towards the LPA with enhanced efficiency (Figure 7B, bottom two lines). Thus when examined in the high levels of detail afforded by our chambers, the growth factors are potentially important accessory factors that increase cell speed and efficiency of chemotaxis, but they do not themselves act as chemoattractants.

These results are reminiscent of observations of development *in vivo*, in which the growth factor SCF promotes migration but not direction of melanoblast migration [46]. It is possible that the melanoma chemotaxis to growth factors observed in other work [13] is due to changes in speed alone, which as discussed earlier can cause a false positive in transwell assays. It has also been shown that growth factors can cause cancer cells to secrete LPA [47], which could also provide an element of indirect chemotaxis in many types of assay.

### LPA Gradients in Tumours *In Vivo*

**Figure 8:**
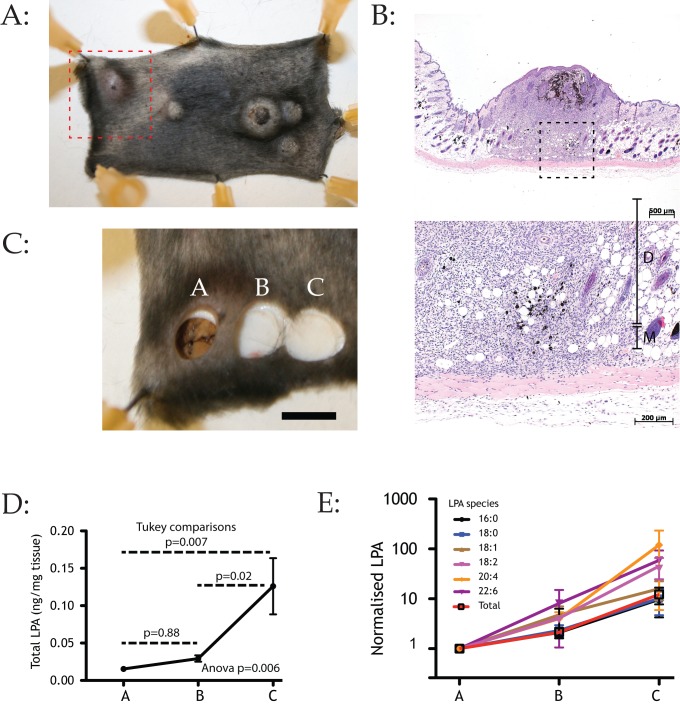
LPA gradients across melanomas *in vivo*. (A) TYR::CreER^T2^BRAF^V600E/+^PTEN^lox/+^ mice, a genetically appropriate melanoma model, were treated with tamoxifen as described in [56], grown until melanomas spontaneously developed. Dashed box shows the region used for the samples shown in Figure 1C. (B) Haematoxylin and eosin-stained biopsies of murine melanomas demonstrating the dispersal of cells from a representative tumour from Figure 5A, with cells spreading directly away from the tumour. Upper image 2.5× magnification; lower image 20× magnification from dashed box above, showing melanoma cells invading toward the muscle layer (D, dermis; M, muscle layer). (C) Biopsies from mouse melanomas. Several sites in a linear distribution were biopsied using a 6 mm punch biopsy tool within 5 minutes of the mouse being sacrificed and immediately frozen in liquid nitrogen. The positions of biopsies used for LPA measurement are indicated (too few distant samples were obtained for a significant measurement). Bar shows 5 mm. (D) LPA concentration gradients across the margin of a melanoma. Four melanomas were sampled at three sites in a line as shown in (A) (A, tumour body; B, tumour edge; C, skin surrounding tumour). Total LPA per mg tissue was quantified by mass spectrometry after weighing the tissue specimens and extracting the LPA. Outward-directed gradients of LPA were found across the margin of all the melanomas tested. Bars show SEM. (E) Analysis of LPA subspecies. 18∶2-LPA, 20∶4-LPA and 22∶6-LPA show a clearer gradient than 16∶0-LPA, which is though to be less active as a signalling molecule.

We have clearly shown that LPA is a potent chemoattractant for melanoma cells of all biological stages. To determine whether this chemotaxis was an important driver of melanoma chemotaxis *in vivo*, we investigated whether the tissue surrounding real melanomas contained LPA gradients that would direct cells out of tumours. Mice that are heterozygotes for the driver mutation Braf^V600E^ (the most prevalent driver of human melanomas) and deletion of the tumour suppressor PTEN develop sporadic melanomas (Figure 8A) genetically and cytologically comparable to human tumours (Figure 8B). We took punch biopsies from the tissue in and across melanomas (Figure 8C) from several mice, extracted total lipids, and examined LPA levels using mass spectrometry. In all non-ulcerated melanomas we examined, LPA levels were low inside the tumour, higher at the edges, and higher still in the tissues immediately outside the tumour (Figure 8D). Cells at the edges of the tumour are therefore experiencing an outward-oriented LPA gradient tending to drive them out into surrounding tissues and vasculature.

We further examined the LPA species in the tissue. Forms that are strongly associated with signalling, in particular 18∶2-LPA and 20∶4-LPA [48], formed the steepest gradients (Figure 8E), while gradients of non-signalling forms such as 18∶0-LPA were flatter. This finding further supports the idea that the gradients of LPA are specifically produced as signals targeted at LPA receptors.

This study is, to our knowledge, the first time a chemotactic gradient has been directly measured around tumours *in vivo*. There are a number of situations where the presence of a gradient has been inferred from cellular behaviour, most prominently in the paracrine loops shown by Segall and others [10]. However, such gradients must by definition be local and tend to be transient. The gradients we observe in melanomas are clear, large-scale, and provide a convincing driver for cell dispersal, and one highly plausible explanation of why melanomas above a certain size, and hence Breslow thickness, always tend to be metastatic.

## Discussion

In this work, we have shown that LPA is a potent chemoattractant for melanoma cells in general, and that outward-oriented gradients of LPA are self-generated by melanoma cells. Because self-generated gradients are always oriented away from tumours, this combination provides a plausible mechanism for driving tumour cell dispersal. We do not exclude other mechanisms; it has for example been proposed that LPA regulates cadherin levels [49], which would not be visible in our assays. Growth factor chemotaxis may be visible under the appropriate conditions (though, as discussed previously, many data are from transwell assays, which are artifact-prone and unreliable). Likewise, we do not exclude other mechanisms than chemotaxis. Contact inhibition of migration occurs in many cell types derived from the neural crest and so is probably found in melanoma, and defects in cell growth and survival in inappropriate locations are of course important factors. But the mechanism we have found that overwhelmingly dominates the dispersal in our assays is robust and is apparently active in a high proportion of melanomas. It is therefore likely to be a particularly important mediator of tumour cell dispersal. We hypothesize that similar mechanisms will be common in cancer metastasis.

### The Source of LPA

The source of LPA around melanomas is unknown. In many tumours, including melanoma, expression of autotaxin and thus autocrine production of LPA has been associated with tumour progression [50]. This LPA production appears to be a mechanism for promoting melanoma growth, rather than driving chemotaxis and invasion. LPA generated by the tumour itself would be found at a higher level in the tumour than outside it, which would oppose outward dispersal and thus metastasis. Rather, we find that the melanoma cells in culture and in tissues break down externally generated LPA, making outward-facing gradients. LPA is therefore more likely to be generated through inflammatory processes—haematopoietic cells, in particular, are a principal source of LPA in tissues [51] —or by inducing LPA production from stromal cells. In metastatic breast cancer xenografts, expression of LPA receptor promotes cell growth and metastasis, but the LPA is made locally by platelets, which are in turn recruited by many tumours [52]. Platelets are also a rich source of growth factors [53]. Our data therefore implicate inflammation in initiating melanoma spread. This finding has important implications for therapy. Interventions that promote inflammation without removing the entire tumour could be extremely dangerous—diagnostic punch biopsies, in particular, could promote a wave of metastasis in response to LPA released by inflammation. From a therapeutic perspective, data from epidemiological studies suggests the anti-inflammatory drug aspirin can protect against metastasis [54].

The increased speed of the metastatic cells may be important, but may also be an artifact of selection. It remains unclear whether the increased speed of migration is clinically important, or whether the fastest strains will metastasize earlier, and thus be the first to be identified. Our data suggest that even less invasive cells move rapidly and accurately enough to metastasize, but our assays may miss factors that retard cell migration.

We have shown that cultured melanoma cells from throughout tumour evolution are chemotactic towards LPA in transwell assays. A recent paper has reported the opposite, that LPA is a chemorepellent for B16 cells [37]. This seems a cell-line specific effect, as these highly derived and divergent cells do not express the LPAR1 and LPAR3 receptors, which are usually highly expressed and dominate LPA chemotaxis in our assays (Figure S2A).

**Figure 9:**
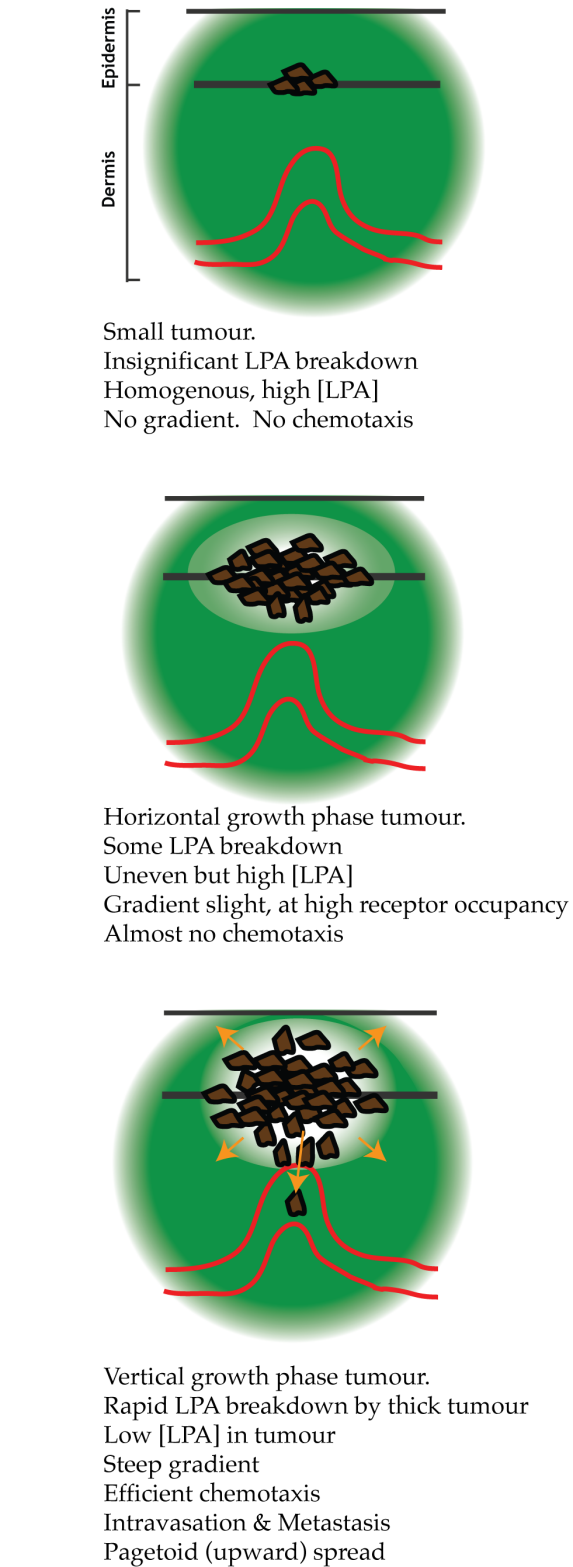
Schematic model of self-generated LPA gradients in melanoma. Like all schematics, this model is intended to clarify the underlying mechanism rather than as a detailed description. In small tumours, the rate of LPA breakdown is insignificant compared to the rates of synthesis and diffusion. Thus although the concentration of LPA is high, there is no gradient, and thus no directional signal. As the tumour becomes thicker—corresponding to an increased Breslow depth—the concentration of LPA at the centre of the tumour drops as the rate of breakdown increases and the distance that LPA must diffuse increases. This generates an LPA gradient that is low inside the tumour and high outside, driving cells to migrate out from the tumour into the surroundings.

We have found that melanomas generate their own chemotactic gradients from homogeneous LPA that is exogenously provided. LPA chemotaxis is an essential feature driving melanoma invasion in 3-D organotypic assays. We have also shown that real tumours create a chemotactic gradient of LPA *in vivo*. Taken together, these lines of evidence suggest a model of chemotaxis towards self-generated LPA gradients is a major driving force for melanoma dispersal (Figure 9). One unforeseen advantage of this model is that it also provides a simple unifying explanation for upward or pagetoid spread, which is a hallmark of the invasive VGP stage melanoma.

### *In Vivo* Models

We have measured actual LPA gradients in animals with experimentally induced melanomas. We have also shown that all the melanoma cells we tested perform chemotaxis towards LPA gradients, in both 2-D and 3-D assays. It is thus reasonable to conclude that LPA gradients are sufficient signals to mediate melanoma cell dispersal.

To test whether LPA is necessary for melanoma metastasis *in vivo* will be very difficult. Our hypothesis is that LPA gradients drive intravasation from the tumour towards local blood vessels. Many widely used metastasis assays, for example tail-vein injection, completely miss this step. Slower assays, for example subcutaneously injected xenografts, metastasize impractically slowly, and to nonphysiological targets, in particular the lymph nodes. Pharmacological approaches, for example blockade of the LPA signalling system by LPA antagonists, are confounded by the importance of LPA to the vascular and haematopoietic systems. A mouse model of melanoma that metastasizes through a physiological route and can be crossed with inducible LPA receptor knockouts does not currently exist; when it is developed, such a model will be the ideal system for testing our model *in vivo*.

### The Gradient, Not the Signal, Is the Information

The most important message from this work is that it is the gradient of LPA—not the presence of LPA *per se*—that contains the information. LPA is a very prevalent molecule. It is present at high levels in serum, and may be generated within tumours by cancer cells or exogenously by, for example, platelet activation. Interestingly, cells ahead of the main group do not respond even when an external gradient is applied (in Movie S3, for example). Presumably these cells reach a region where LPA levels are homogeneously high, at which point there is little or no guidance information available to them. Likewise, if too few cells are used in the spread cell assay, no chemotaxis is observed, suggesting that LPA breakdown is important even in classical chamber assays. We suspect that LPA is not a chemoattractant for low densities of cells, because they cannot break it down rapidly enough to form an appropriate local gradient. In our invasion assays, LPA becomes an attractant when—counterintuitively—cells are present at high enough densities to break down most of it. This means that the LPA gradient is self-generated by the melanoma.

Self-generated gradients are currently highly topical. Recent papers showing the detailed roles of the CXCR4 and CXCR7 receptors (which respond to and deplete SDF-1, respectively) during the formation of the zebrafish lateral line have caused a spike in interest, but other methods whereby cells drive creation of attractant gradients then respond to them occur in multiple systems, especially during embryonic development [26],[27],[55],[56]. More generally self-generation provides a means whereby cells can maintain a directional cue over distances that are far too large for premade gradients. Furthermore, with externally formed gradients, the information that specifies the gradient must come from somewhere else. If an external gradient attracts cells during development, the secret to understanding the process lies with understanding where and by whom the attractant is being made. Self-generated gradients are different; there is no need for external information. The gradient is generated as an emergent property of the interaction between the cells and their environment. Thisconclusion is perhaps the most interesting feature of this work. In LPA chemotaxis during melanoma metastasis, there is no need for any other cell type to set up a local gradient. The melanoma cells first generate a gradient—once the tumour is thick enough—and then respond to it by migrating away. Thus the melanoma drives its own metastasis.

## Methods

### Ethics

All mice used were control cohorts from other studies. Before they were humanely killed, all mice had reached the primary or secondary end-points of their designated study.

### Cell Lines

All melanoma cell lines used are listed by biological stage of derivation and were transferred from the Wellcome Trust Functional Genomics Cell Bank (Biomedical Sciences Research Centre, St. George's, University of London).

Cells were maintained in Roswell Park Memorial Institute (RPMI, Invitrogen) 1640 medium, supplemented with 10% fetal bovine serum (FBS) (PAA Labs), 2 mM L-Glutamine (Gibco, Invitrogen), and 1% penicillin and streptomycin (Gibco, Invitrogen).

siRNA constructs were obtained from QIAGEN and transfected as per instructions. WM239A cells were challenged twice with siRNA, 48 hours apart, then used in the assay 48 hours after the second transfection.

### Insall Chamber Chemotaxis Assay

Insall chambers were manufactured and used as described [15]. The chambers were drilled in advance with a 1.3 mm drill bit using an overhead drill press. During drilling, the chamber was secured in a small machine vice sitting inside a V-block at 45° and a hole was drilled into each “rabbit ear” of the outer well to allow reverse filling. Cells were starved in PBS for 12 hours then seeded at a density of 5.5×10^4^ cells/ml in CGM. Each cover slip was coated with 2 ml of the seeding suspension. After seeding cells, the six-well dish was shaken in the *x* and then *y* planes for 5 seconds each and placed in a CO_2_ incubator at 37°C on top of a shock absorbent base to prevent vibration induced patterns of cell accumulation.

VALAP sealant (vaseline, lanolin, and paraffin) was prepared by combining the three components together in a weight ratio 1∶1∶1 and melting at 100°C on a heat block. A fine artist's paint brush was used to apply the VALAP.

Cover slips were treated with human fibronectin (BD Biosciences) 1 mg/ml throughout, generating an adsorbed concentration of 4.17 µg/cm^2^ in the range of 1–5 µg/cm^2^ as suggested by the manufacturer. Following fibronectin coverslips were passivated with 0.5% (w/v) heat-treated BSA solution in PBS.

Chemoattractants were added to serum-free RPMI medium as required. Addition of 5 mM HEPES to the media in the sealed chamber is essential to buffer the pH of the media throughout the experiment. LPA (Sigma) was dissolved in a 1∶1 ratio of distilled water: absolute ethanol to generate a 1 mM stock solution and stored at −20°C. To use this as a chemoattractant, BSA was diluted to a final concentration of 0.05% (w/v) to SFM-H (SFM-HB) and then 1 µl LPA was added to 1 ml to generate a 1 µM LPA solution. EGF (Peprotech), PDGF, BB Homodimer (Calbiochem), HGF/Scatter Factor (Peprotech), and SDF-1α/CXCL12 (Peprotech) were dissolved in PBS to a stock concentration of 10–100 µg/ml, stored at −20°C and used as indicated.

Ki16425 (Cambridge Bio) was stored in absolute ethanol at a stock concentration of 10 mM as per the manufacturer's instructions. In Insall chamber assays, cells were pre-incubated for 5 minutes with a 10 µM solution before combining with reagents in the chamber at the same concentration.

### Time-lapse Microscopy

We used a Nikon TE2000-E inverted time-lapse microscope equipped with a motorised stage (Prior) and Perfect Focus System (PFS) to prevent focal drift due to thermal fluctuations. The entire microscope was enclosed in a plexiglass box, humidified and maintained at 37°C with 5% CO_2_. The Insall chamber experiments did not require the addition of supplementary CO_2_. Our microscope system was driven by Metamorph software (Molecular Devices) and the x, y positions were manually selected and pre-loaded.

### Image Processing

Images were processed using ImageJ (http://rsb.info.nih.gov/ij/), if necessary using the Image stabilizer plugin (http://www.kangli.org/code/Image_Stabilizer.html) to correct for drift. Cells were tracked using MtrackJ (http://www.imagescience.org/meijering/software/mtrackj/) to follow the path of the cell nucleus For consistency, we attempted to track a minimum of 40 cells in every chamber assay; in most cases this was sufficient to ensure statistical significance. The following criteria were used for deciding which cells to track: cells that moved more than 1 cell length in 24 hours; cells that tracked continuously until the end of the experiment or until the cell migrated off the bridge or rounded up in preparation for mitosis; cells were excluded that migrated onto the bridge during the experiment; avoided tracking post-mitotic cells.

We developed an Excel spreadsheet (written by DMV and AJM-M) to facilitate the processing, analysis, and quantification. This spreadsheet automatically produces spider plots, speed, and chemotaxis index data over time. A time window was selected (e.g., 6–12 hours for melanoma cells) and values zeroed within this window to produce end-point data. Chemotaxis index (cosθ) plots are presented as mean ± standard error of the mean (SEM). Cosθ is a function of the distance migrated in the direction of the gradient divided by the euclidian distance (the linear distance between the start and end position of the cell). These data were also processed in the Circstat toolbox for MATLAB by GK [30]. This process generated rose and polar plots with 95% confidence intervals and a Rayleigh test.

### Conditioned Media Preparation for Chemotaxis Assays

Conditioned media were generated as follows. A sub-confluent 10 cm petri dish of WM239A cells was washed 3× with PBS then cells were split in a 1∶5 ratio into five new 10 cm petri dishes and combined with fresh CGM to a final volume of 10 ml. Conditioned medium was then harvested from one dish per time-point, staggered between 0–48 hours (Marked T0, T6, etc.). All 10 ml was aliquoted into 10×1 ml eppendorf tubes. The samples were immediately frozen on dry ice before storing at −80°C. The cells in each dish were then counted. When needed aliquots of conditioned media were thawed at 37°C and centrifuged for 10 min using a lab top centrifuge, then filtering with a sterile 0.2 µm filter.

### Organotypic Invasion Assay

Collagen gels were prepared by combining 2 mg/ml rat tail collagen solution, 10× Minimum Essential Medium (MEM, Invitrogen), and 0.22 M NaOH in a ratio 8∶1∶1. The pH was finely adjusted to pH 7.2 with the 0.22M NaOH. One volume of FBS containing 7.5×10^5^ primary human skin fibroblasts (passage 5–7) was immediately combined with 10 ml of the gel mixture on ice. After pipetting well, 2.5 ml of the gel and cell mixture was added to each 35 mm petri dish. The gels were then placed in a humidified incubator with 5% CO_2_ to set for 15–30 minutes. A further 1 ml MEM was added to each petri dish and the gels were carefully detached to enable gel contraction in the same incubator. The media was changed every 3 days. After 6–7 days the gels measured approximately 1.5 cm in diameter and were transferred to a 24-well dish ready for tumour cell seeding. 1–2×10^5^ tumour cells were then counted and allowed to seed on the surface of each gel. The gel was carefully transferred with forceps to an elevated stainless steel grid (Sigma, screens for CD-1, size: 40 mesh) and placed in a 6 cm petri dish and this was denoted day 0. CGM was added to cover the grid and was then carefully aspirated to leave a meniscus around the base of each gel, thereby generating an air-liquid interface. Three gels were loaded onto each grid and the medium was changed three times weekly. In experiments using Ki16425, the gels with adherent cells were pre-incubated for 5 minutes with 10 µM Ki16425 in the CGM before raising the gels to the air-liquid interface. 10 µM Ki16425 was maintained in the CGM throughout the experiment with thrice weekly media changes as before. A typical experiment lasted 7–12 days.

At the end of the invasion assay, each gel was divided into two with a scalpel, fixed in 4% formaldehyde at 4°C and sectioned before being stained with haematoxylin and eosin.

### Murine Melanoma Tissue

We used the inducible Tyr::CreER^T2^ BRAF^V600E/+^ PTEN^lox/−^ melanoma model [57], in which the melanomas were all generated in mixed background mice from 6–12 weeks of age. Animals were treated with 2 mg tamoxifen topically to shaved back skin daily for 5 days. There was no discernable phenotype until naevi or primary melanomas started developing 6–8 weeks after induction predominantly on the treated area. Typical grooming behaviour spread the tamoxifen to other parts of the skin and/or was ingested leading to activation in other cutaneous regions. All mice used were control cohorts from other studies. Before they were killed, all mice had reached the primary or secondary end-points of their designated study.

Suitable mice were identified with at least one and up to four tumours, ideally located on the back. The smallest tumour size was 4×4 mm to enable at least two areas to be sampled. Skin containing the tumours was rapidly dissected off the back and pinned slightly taut to paper overlying a corkboard. Sterile Punch Biopsy tools (Stiefel) were used to punch circular samples from the tumour and surrounding skin. The size of punch biopsy depended on the tumour size and varied from 3–6 mm in diameter. Samples were taken at various locations across the tumour and were coded as follows: within the tumour (A), across the margin (B), 5 mm from the margin (C), and 10 mm from the margin (D). Samples were immediately snap frozen in liquid nitrogen and transferred to a −80°C freezer for storage.

Control samples of normal appearing skin in the same melanoma model activated with tamoxifen were used to calculate the basal level of LPA. Each section of mouse skin underwent a series of nine punch biopsies (A, B, and C in three replicate series).

### Liquid Chromatography-Mass Spectrometry

Mice and human melanoma/skin samples (1–20 mg) were pulverised after thoroughly cooling with liquid nitrogen. The pulverised powder was suspended in 750 µl water then used for LPA extraction. For cell culture media samples, 750 µl of cell culture media was used for LPA extraction. Media or tissue samples were spiked with 50 ng of 17∶0-LPA as an internal standard before extraction. LPA was extracted with 1 ml n-butanol three times at room temperature.

The combined LPA extract was dried under vacuum with SpeedVac (Thermo) and re-dissolved in 60 µl chloroform/methanol/water 2∶5∶1. 14 µl was injected for liquid-chromatography with tandem mass spectrometry (LC-MS/MS) analysis. For LC-MS/MS analysis, we used a Thermo Orbitrap Elite system (Thermo Fisher) hyphenated with a five-channel online degasser, four-pump, column oven, and autosampler with cooler Shimadzu Prominence HPLC system (Shimadzu) for lipids analysis. High resolution/accurate mass and tandem MS were used for molecular species identification and quantification. The identity of the lipid subspecies was further confirmed by reference to appropriate lipids standards. All the solvents used for lipid extraction and LC-MS/MS analysis were LC-MS grade from Fisher Scientific.

The final amount of LPA (ng) is presented as a concentration per 750 ml of conditioned media analysed or per mg tissue. The data are represented graphically plotting mean ± SEM for the concentration of LPA versus conditioning time (for conditioned media samples); and distance from tumour margin (for tumour samples). Samples were normalised to position “A” for comparison between tissue samples.

## Supporting Information

Figure S1 **Paths of cells with and without cell∶cell contacts.** Distances are shown in microns. Cells that are contacting one or more other cells are represented as red dots. Cells that are moving without cell∶cell contacts are represented as green dots. There is no visible difference in directional accuracy or speed between the cells with and without contacts.
(PDF)

Figure S2 **Inhibition of LPA chemotaxis.** (A) Serum chemotaxis is blocked by siRNA inhibition of LPAR1. Assays were performed exactly as in Figure 1G, using WM239A cells that had been transfected with a non-silencing RNA (left) or siRNA against LPA Receptor 1 (LPAR1; Qiagen flexitube GeneSolution, catalogue number GS1902; right). (B) LPA chemotaxis is blocked by LPA receptor antagonists. WM1158 cells were assayed as described for Figure 4D, in the presence of the LPAR1/3 antagonist Ki16425 (right panel) or a comparable amount of ethanol vehicle (left panel).
(PDF)

Figure S3 **3-D organotypic assay performed while collagen plugs remained submerged in medium.** The cell line WM98-1 that is highly chemotactic towards serum in 3-D organotypic assays, fails to perform chemotaxis if the gels are kept submerged throughout the 14 day assay period, despite growing on top of the plug.
(PDF)

Movie S1 **Outward migration of densely packed melanoma cells in the absence of a gradient.** Both wells are filled with complete medium containing 10% FBS, but WM239A cells are only inoculated in the left well. See Figure 1A for details. Time stamps and scale bar are shown for reference.
(MOV)

Movie S2 **Outward migration is not dependent on contact inhibition.** See Figure 1D for details. WM1158 cells chemotax equally effectively whether or not they are contacting their neighbours. Time stamps and scale bar are shown for reference.
(MOV)

Movie S3 **Outward migration up a serum gradient.** WM239A cells are inoculated in the left well in medium without serum, then the right well was filled with medium containing 10% FBS. See Figure 1G for details. Time stamps and scale bar are shown for reference.
(MOV)

Movie S4 **Effects of the LPA inhibitor Ki16425 on density-dependent dispersal.** The movie shows two experiments, without (left) and with (right) 10 µM Ki16425. In each case both wells contains medium with 10% FBS as in Movie S1. Cells were introduced into the left lane at time zero. Time stamps and scale bar are shown for reference.
(MOV)

Movie S5 **Effects of the LPA inhibitor Ki16425 on serum chemotaxis.** The movie shows two experiments, without (left) and with (right) 10 µM Ki16425. WM239A cells were spread evenly on coverslips in chemotaxis chambers. In each case the left hand well contains medium without serum and the right hand well contains medium with 10% FBS. See Figure 4A for details. Time stamps and scale bar are shown for reference.
(MOV)

## Abbreviations

EGF: epidermal growth factor
FBS: fetal bovine serum
LPA: lysophosphatidic acid
PDGF: platelet-derived growth factor
RGP: radial growth phase
SCF: stem cell factor
SEM: standard error of the mean
VGP: vertical growth phase
