# Supplementary figures and images for: Melanoma Cells Break Down LPA to Establish Local Gradients That Drive Chemotactic Dispersal
Source: PLoS Biol. 2014 Oct 14;12(10):e1001966. doi: 10.1371/journal.pbio.1001966 (PMC4196730; doi:10.1371/journal.pbio.1001966)

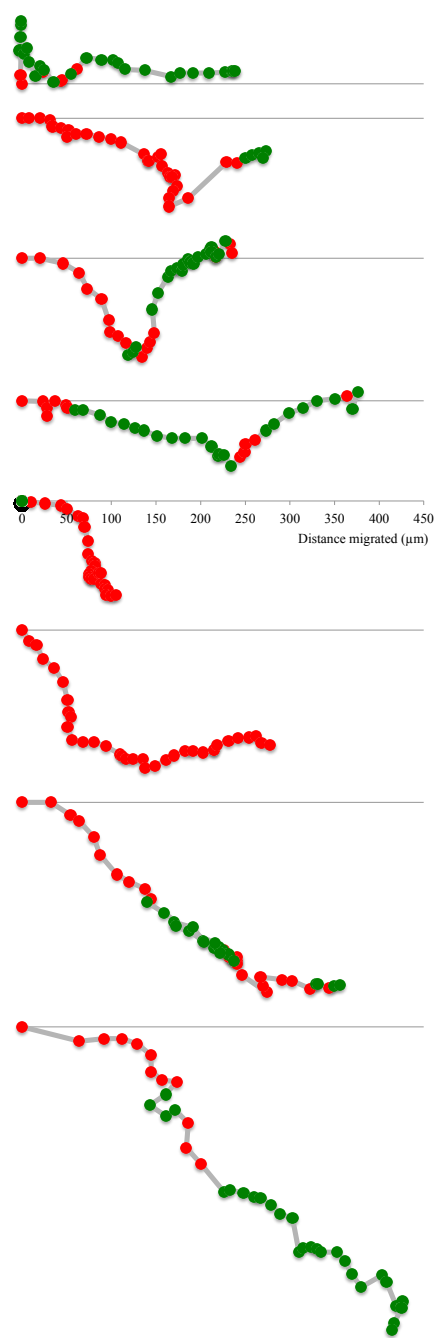

Supplement: Figure S1 — Paths of cells with and without cell∶cell contacts. Distances are shown in microns. Cells that are contacting one or more other cells are represented as red dots. Cells that are moving without cell∶cell contacts are represented as green dots. There is no visible difference in directional accuracy or speed between the cells with and without contacts. (PDF) [file pbio.1001966.s001.pdf]

A:

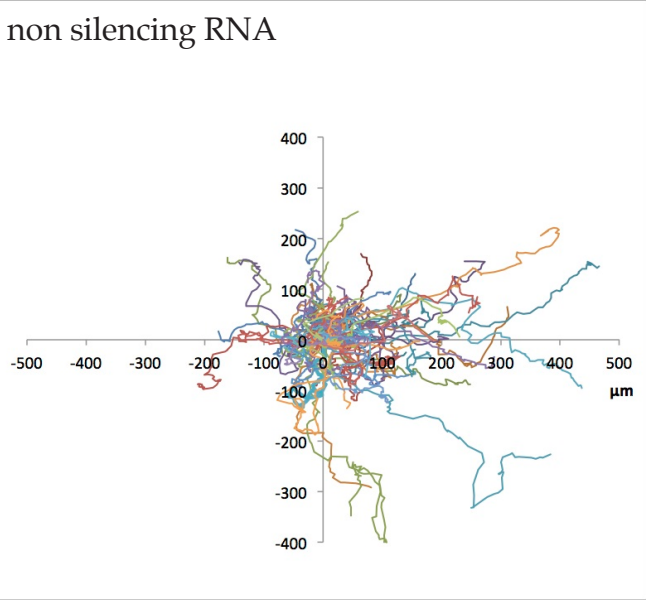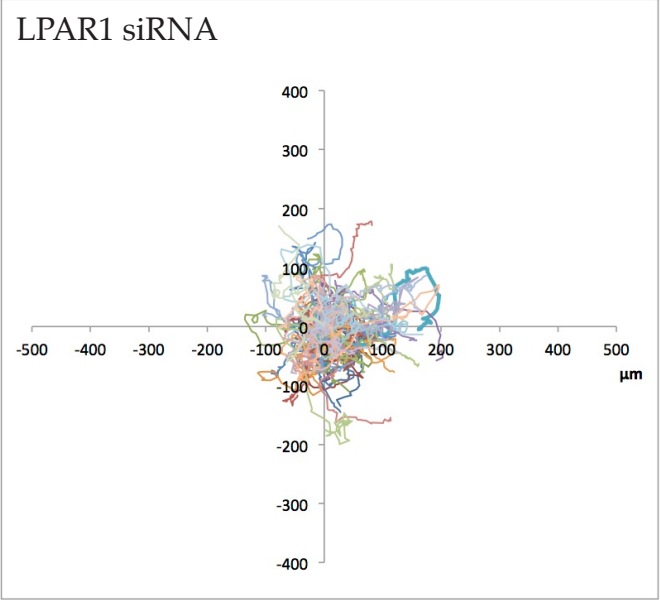

B:

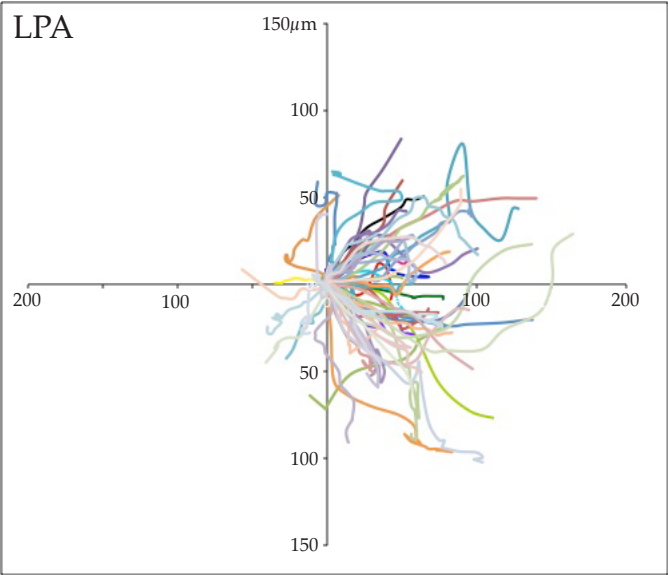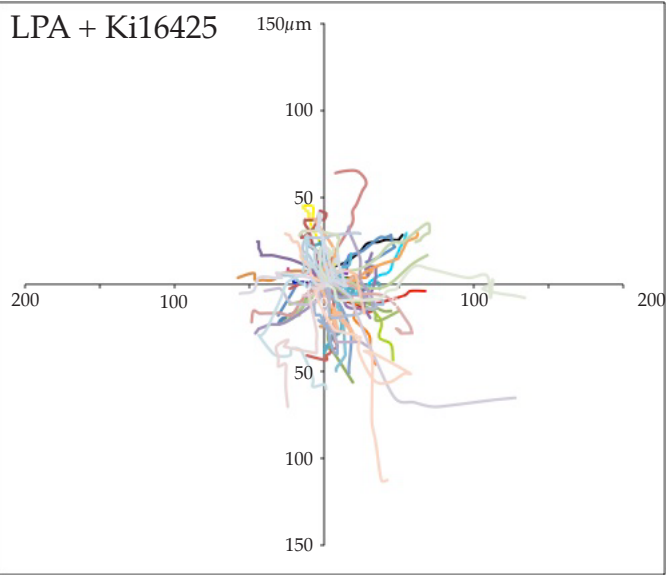

Supplement: Figure S2 — Inhibition of LPA chemotaxis. (A) Serum chemotaxis is blocked by siRNA inhibition of LPAR1. Assays were performed exactly as in Figure 1G, using WM239A cells that had been transfected with a non-silencing RNA (left) or siRNA against LPA Receptor 1 (LPAR1; Qiagen flexitube GeneSolution, catalogue number GS1902; right). (B) LPA chemotaxis is blocked by LPA receptor antagonists. WM1158 cells were assayed as described for Figure 4D, in the presence of the LPAR1/3 antagonist Ki16425 (right panel) or a comparable amount of ethanol vehicle (left panel). (PDF) [file pbio.1001966.s002.pdf]

# Muinonen-Martin et al. Supplementary figure 3

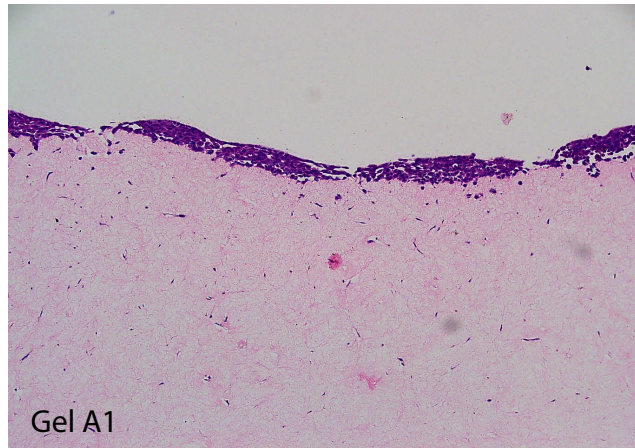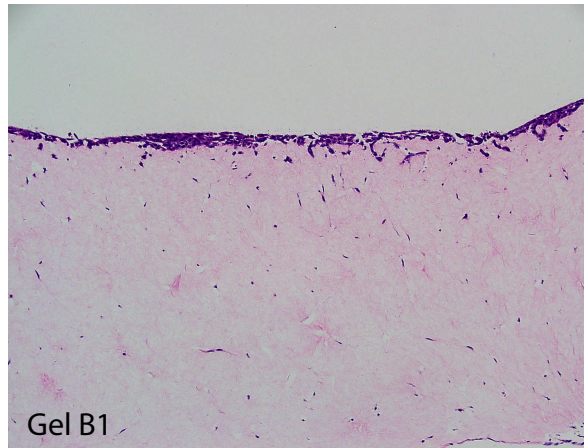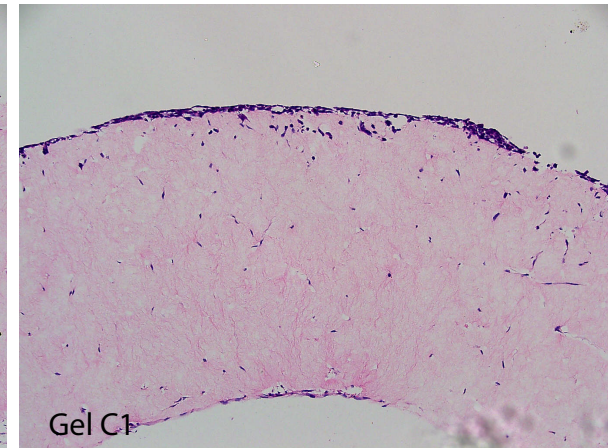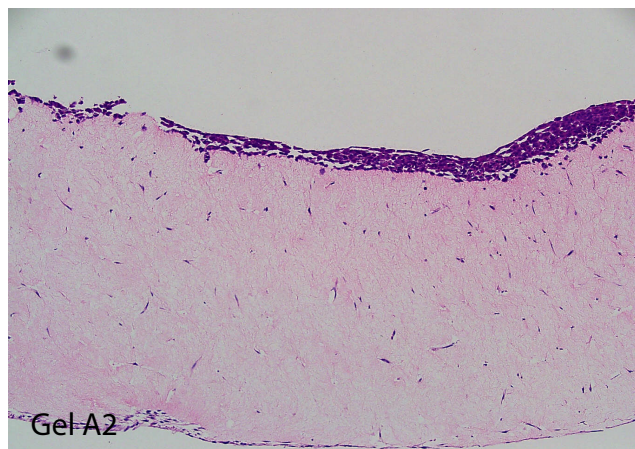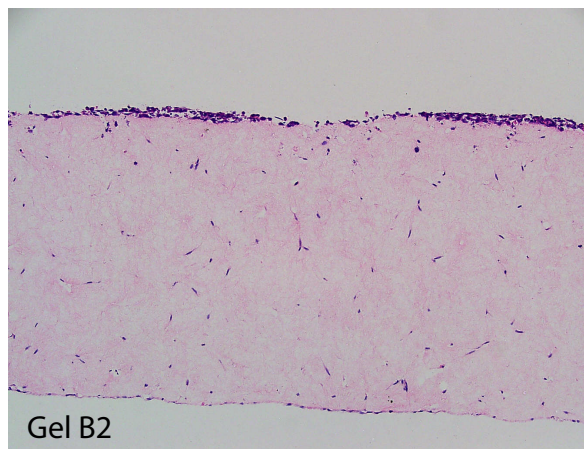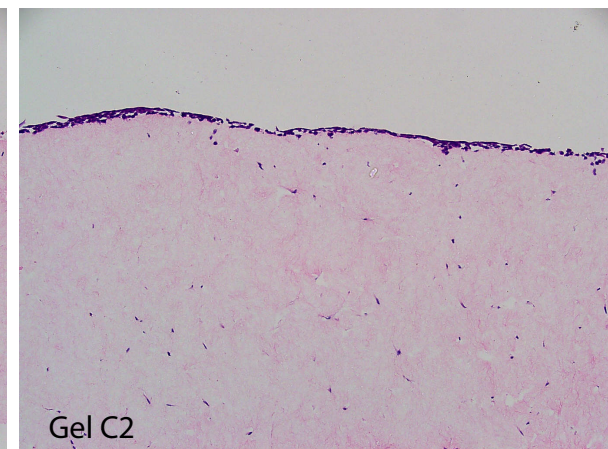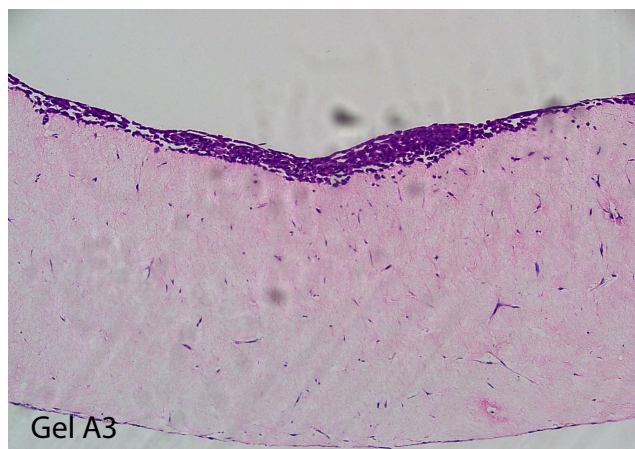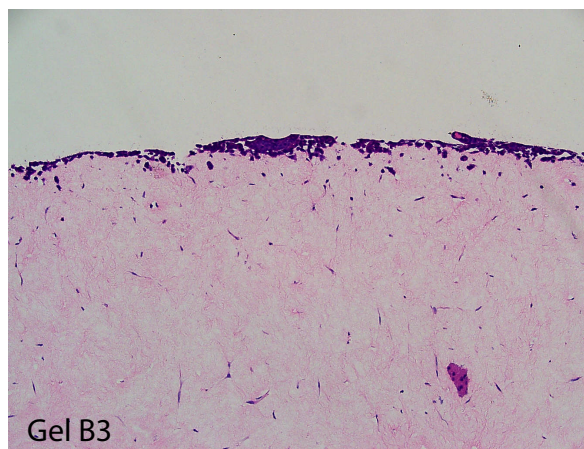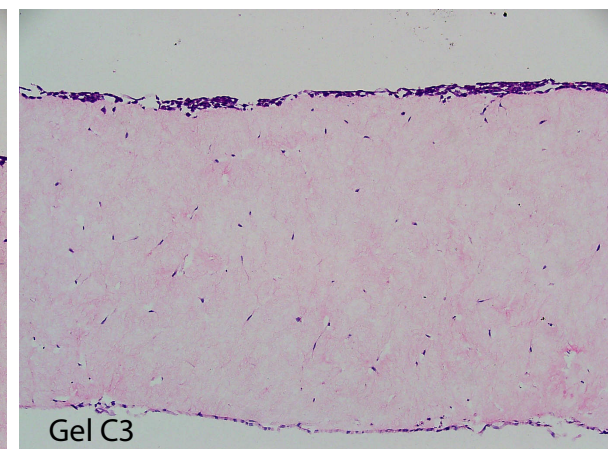

Supplement: Figure S3 — 3-D organotypic assay performed while collagen plugs remained submerged in medium. The cell line WM98-1 that is highly chemotactic towards serum in 3-D organotypic assays, fails to perform chemotaxis if the gels are kept submerged throughout the 14 day assay period, despite growing on top of the plug. (PDF) [file pbio.1001966.s003.pdf]
